# Histone H1x is highly expressed in human neuroendocrine cells and tumours

**DOI:** 10.1186/1471-2407-8-388

**Published:** 2008-12-24

**Authors:** Julia Warneboldt, Florian Haller, Olaf Horstmann, Bernhard C Danner, László Füzesi, Detlef Doenecke, Nicole Happel

**Affiliations:** 1 Institute for Biochemistry and Molecular Cell Biology, University of Göttingen, Humboldtallee 23, D-37073 Göttingen, Germany; 2 Department of Gastroenteropathology, University Medicine Göttingen, Robert-Koch-Str. 40, D-37099 Göttingen, Germany; 3 Department of General- and Visceral Surgery, University Medicine Göttingen, Robert-Koch-Str. 40, D-37099 Göttingen, Germany; 4 Department of Cardiothoracic and Vascular Surgery, University Medicine Göttingen, Robert-Koch-Str. 40, D-37099 Göttingen, Germany; 5 Sana Kliniken Düsseldorf GmbH, Gräulinger Str. 120, D-40625 Düsseldorf, Germany

## Abstract

**Background:**

Histone H1x is a ubiquitously expressed member of the H1 histone family. H1 histones, also called linker histones, stabilize compact, higher order structures of chromatin. In addition to their role as structural proteins, they actively regulate gene expression and participate in chromatin-based processes like DNA replication and repair. The epigenetic contribution of H1 histones to these mechanisms makes it conceivable that they also take part in malignant transformation.

**Methods:**

Based on results of a Blast data base search which revealed an accumulation of expressed sequence tags (ESTs) of H1x in libraries from neuroendocrine tumours (NETs), we evaluated the expression of H1x in NETs from lung and the gastrointestinal tract using immunohistochemisty. Relative protein and mRNA levels of H1x were analysed by Western blot analysis and quantitative real-time RT-PCR, respectively. Since several reports describe a change of the expression level of the replacement subtype H1.0 during tumourigenesis, the analysis of this subtype was included in this study.

**Results:**

We found an increased expression of H1x but not of H1.0 in NET tissues in comparison to corresponding normal tissues. Even though the analysed NETs were heterogenous regarding their grade of malignancy, all except one showed a considerably higher protein amount of H1x compared with corresponding non-neoplastic tissue. Furthermore, double-labelling of H1x and chromogranin A in sections of pancreas and small intestine revealed that H1x is highly expressed in neuroendocrine cells of these tissues.

**Conclusion:**

We conclude that the high expression of histone H1x in NETs is probably due to the abundance of this protein in the cells from which these tumours originate.

## Background

The nuclear DNA of eukaryotic cells is organised as chromatin in association with proteins. The basal structural organisation unit of chromatin is the nucleosome consisting of the nucleosome core particle, linker DNA and histone H1. The nucleosome core particle is composed of two molecules of each of the four core histones which form an octamer around which DNA with a length of 146 base pairs is wrapped. These nucleosome cores are connected by linker DNA of variable length. H1 histones are positioned at the linker DNA between the nucleosome cores and therefore they are also called linker histones [1,2]. To date, eleven H1 homologous proteins have been described in humans, including the ubiquitously expressed subtype H1x [3]. Comparison of the biochemical behaviour of H1x with other H1 subtypes revealed similarities but also showed characteristic variations [4]. The regulation of the *H1x *gene expression differs from that of the replication-dependent main class subtypes, but also from that of the replacement subtype H1.0. Interestingly, the activity of H1x seems to be controlled not only on level of expression, but also by a cell-cycle-dependent change of its intranuclear distribution [5].

H1 histones actively regulate chromatin processes such as gene expression [6], DNA replication [7] and repair [8]. Aberrations in such epigenetic mechanisms can be associated with malignant transformation [9,10]. Since histone H1 modulates both chromatin structure and transcriptional activity, it is conceivable that H1 histones may contribute to epigenetic phenomena leading to malignant transformation. Change of the H1 subtype composition during tumourigenesis has been described in several studies [11-13]. A greater number of these reports deals with the replacement H1 subtype H1.0. Some of these data showed an increase of H1.0, others demonstrated a decrease in comparison to the paired normal tissue [14,15]. During our studies on characteristics of the H1 subtype H1x, a Blast data base search revealed an accumulation of expressed sequence tags (ESTs) of H1x in libraries from neuroendocrine tumours (NETs). NETs are a very heterogenous group of rare epithelial tumours that originate from neuroendocrine cells and mostly occur in the gastrointestinal tract and in the lung [16-18]. In the present study we investigated the occurrence of H1x in cells of NET tissues using immunohistochemistry and compared the expression of H1x in NETs with that in paired normal tissue on protein and on mRNA level using Western blot analysis and quantitative real-time RT-PCR, respectively. Furthermore, double labelling of H1x and chromogranin A, a marker for neuroendocrine cells, of sections from normal tissue of pancreas and small intestine revealed that H1x is highly expressed in neuroendocrine cells of these tissues.

## Methods

### Bioinformatics

Data base searches were carried out with the Blast program .

### Tumour samples

Snap-frozen as well as formalin-fixed and paraffin-embedded tumour samples from 13 primary NETs and from 1 liver metastasis of a NET were recruited. Paired non-neoplastic tissues of 8 tissue samples were used as control (Table 1). Pathohistological examination was performed using haematoxylin-eosin-stained sections of each specimen. The study was performed according to the ethical guidelines of the Declaration of Helsinki and was approved by the local ethics committee (no. 32/12/05).

**Table 1:** Tumour samples from neuroendocrine tumours (NETs) and paired non-neoplastic tissue samples used in the present study.

**Patient no**.	**Sample no**.	**Organ**	**Tissue**	**Subtype**
1	1	lung	non-neoplastic	

1	2	lung	NET	Carcinoid

2	3	lung	non-neoplastic	

2	4	lung	NET	poorly differentiated small cell carcinoma

3	5	lung	non-neoplastic	

3	6	lung	NET	carcinoid

4	7	lung	NET	poorly differentiated small cell carcinoma

5	8	small intestine	non-neoplastic	

6	9	small intestine	non-neoplastic	

7	10	small intestine	NET	well-differentiated carcinoma

8	11	small intestine	NET	poorly differentiated small cell carcinoma

9	12	small intestine	NET	well-differentiated carcinoma

10	13	small intestine	NET	carcinoid

11	14	small intestine	NET	carcinoid

12	15	pancreas	non-neoplastic	

13	16	pancreas	non-neoplastic	

13	17	pancreas	NET	well-differentiated carcinoma

14	18	pancreas	NET	poorly differentiated large cell carcinoma

15	19	pancreas	NET	well-differentiated carcinoma

16	20	pancreas	NET	well-differentiated carcinoma

17	21	liver	non-neoplastic	

17	22	liver	NET	liver metastasis of well-differentiated carcinoma of pancreas

### Immunohistochemistry

For immunohistochemical analysis, tissue microarrays (TMAs) were constructed using a semi-automated manual tissue arrayer (Alphametrix, Rodgau, Germany) to conduct tissue punches with a diameter of 1 mm. For each of the samples, 6 tissue punches from different areas were analysed. The 1 μm thick sections were mounted on sialanised slides, dewaxed, rehydrated and antigen-retrieved in 10 mM citrate buffer (pH 6.0) for 45 min in a pressure cooker. The following steps were carried out using the Dako Cytomation ChemMate Detection Kit (Dako, Glostrup, Denmark) according to the manufacturer's description. The sections were probed with affinity-purified anti-H1x antibody at a final concentration of 1.5 μg/ml [5] or anti-chromogranin A antibody (DAK-A3, Dako, Glostrup, Denmark) in a 1:150 dilution. The slides were counterstained with aniline blue and mounted in xylol.

### Double-labelling experiments

Sections from formalin-fixed and paraffin wax-embedded samples were deparaffinised in xylol, rehydrated, and washed in Tris buffered saline (TBS; 50 mM Tris, pH 7.5, 152 mM NaCl). Antigen-retrieval was done by incubation in 10 mM citrate buffer (pH 6.0) for 45 min at 95°C. Sections were permeabilised with 0.3% Triton X-100 in TBS for 45 min and blocked by incubation with 3% BSA in TBS containing 0.05% Tween-20 (TBS-T). Sections were then incubated with the following primary antibodies: Affinity-purified anti-H1x antibody at a final concentration of 1.5 μg/ml [5] or anti-chromogranin A antibody (DAK-A3, Dako, Glostrup, Denmark) in a 1:150 dilution. After washes in TBS (3 × 5 min), sections were incubated with the following secondary antibodies in a 1:1000 dilution: Alexa-Fluor 555-anti-mouse IgG (#21425), Alexa Fluor 488-anti-rabbit IgG (#11070) from Molecular Probes. Thereafter, sections were rinsed in TBS (3 × 5 min), dehydrated and cleared in xylol. The nuclei were visualised with the fluorochrome 4'-6-diamidine-2-phenyl indole (DAPI) using Vectashield Mounting Medium with DAPI (Vector Laboratories, Burlingame, USA). For fluorescence microscopy an Axioskop (Zeiss, Göttingen, Germany) was used.

### Histone extraction

Purification of whole histones from snap-frozen tissue samples was done using sulfuric acid. 100 mg tissue was homogenised in 1 ml PBS, containing protease inhibitors (Complete, Roche Diagnostics, Mannheim, Germany) using an ultra turrax. Sulfuric acid was added (final concentration 0.2 M) to the homogenate, and histone extraction was done by incubation for one hour on ice. Samples were centrifuged (10 min, 4°C, 14,000 × g) and the acid-soluble proteins in the supernatant were precipitated with a final concentration of 20% TCA (w/v) for one hour. After centrifugation (30 min, 4°C, 14,000 × g), the pellet was washed with cold (-20°C) acetone and air-dried. The histones were dissolved in 30 mM HCl.

### Immunoblotting

Histones were separated on 15% SDS-polyacrylamide gels and electrophoretically transferred to nitrocellulose. Uniform blotting was checked by staining the nitrocellulose filter with Ponceau S (Sigma, Taufkirchen, Germany). Blots were probed with polyclonal H1x-specific antiserum in a 1:5000 dilution [4], anti-H1.2 antibody (#ab17677, Abcam, Cambridge, UK) in a 1:1000 dilution or a mouse monoclonal anti-H1.0 antibody (kindly provided by Prof. H. Zentgraf, German Cancer Research Center, Heidelberg, Germany) in a 1:160 dilution. The immunoreactive proteins were visualized using the chemiluminescence ECL plus detection system (Amersham Bioscience, Freiburg, Germany) after incubation with a horseradish peroxidase-conjugated goat anti-rabbit antibody (#A0545, Sigma, Taufkirchen, Germany) or horseradish peroxidase-conjugated goat anti-mouse antibody (#A9917, Sigma, Taufkirchen, Germany). Stripping of blotting membranes for reprobing was done by incubation of the membranes in stripping buffer (100 mM 2-Mercaptoethanol, 2% SDS, 62.5 mM Tris-HCl, pH 6.7) for 30 min at 50°C with occasional agitation. Bands on the ECL film were quantified with the 1Dscan Ex gel analysis software (Scanalytics, Rockville, USA). The ratios of H1x and H1.0 to H1.2 were calculated, and the mean values of the non-neoplastic tissues were set to 1 to show the fold-change difference of the protein amounts in the NETs.

### Quantitative real-time RT-PCR

Total RNA was extracted from snap-frozen tissue material with TRIzol reagent (Invitrogen Life Technologies, Mannheim, Germany) according to the manufacturer's manual and dissolved in RNase-free water. Total RNA concentration was quantified with the RNA 6000 nano LabChip using the Agilent 2100 Bioanalyzer (Agilent Technologies, Palo Alto, USA). Superscript II RNase H- Reverse Transcriptase (Invitrogen Life Technologies) was used to generate first strand cDNA from 5 μg RNA per sample according to the manual. 125 ng of random hexamer primers (Invitrogen Life Technologies) and 40 U of RNasIn ribonuclease inhibitor (Promega, Mannheim, Germany) were applied per sample. H1 subtype expression was analyzed with quantitative real-time RT-PCR using gene-specific primers (H1x, primer A, CCCAACGATGTAGCGTTTTT, primer B, AAGGCCGAGAGCCAATAGA, amplicon length, 80 bp; H1.2, primer A, GCCACTTGTACCCGAGTTTT, primer B, TTCTTCTTTACAGGGGCCTTC, amplicon length, 99 bp). Triplicate PCRs were run in an iCycler (Bio-Rad Laboratories GmbH, München, Germany) using the Eurogentec qPCR Core kit for SYBR Green I No ROX (Eurogentec, Seraing, Belgium) and gene-specific primers in a final concentration of 300 nmol/l. The temperature profile consisted of (i) an initial step at 95°C for 10 min; (ii) 40 cycles at 95°C for 15 s and 60°C for 60 s; and (iii) a final melting curve analysis with a temperature ramp from 60 to 95°C and a heating rate of 3°C/min. PCR efficiencies were calculated with a relative standard curve derived from a cDNA mixture (a twofold dilution series with seven measuring points in triplicates) and gave regression coefficients >0.95 and reproducible primer-specific efficiencies of 84–99%. Gene-specific amplification was confirmed by a single peak in melting curve analysis and a single band in agarose gel electrophoresis. No template controls (no cDNA in PCR) and genomic controls (no enzyme in reverse transcription) were run for each gene to detect unspecific and genomic amplification or primer dimerisation. Gene expression levels were determined by relative quantification using the expression of histone *H1.2 *for normalization. To compare the Ct-values of the qRT-PCR results from different samples, the 2^-(ΔΔCt) ^method described by [19] was applied. Statistics, Wilcoxon test and graphs were performed with Statistica 6.0 (StatSoft, Hamburg, Germany).

## Results and discussion

### Immunohistochemical detection of H1x in NETs

**Figure 1:**
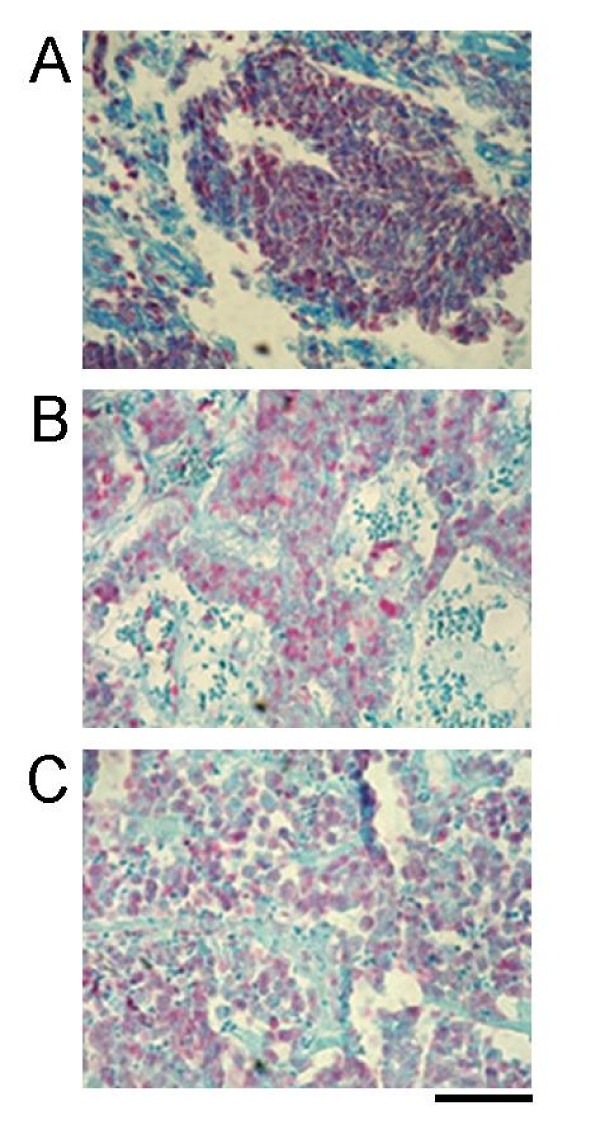
**Immunohistochemical detection of histone H1x in sections from NET tissues**. The three different NETs were (A) poorly differentiated carcinoma of lung (Table 1, sample no. 4), (B) well-differentiated pancreatic carcinoma (sample no. 19) and (C) poorly differentiated carcinoma of small intestine (sample no. 11). The tumour cells showed an intensive immunoreaction with the anti-H1x antibody. Scale bar = 100 μm.

A Blast data base search revealed a strikingly high portion of EST entries of the H1 subtype H1x from the libraries LU24 and LU5 (225 entries of 500 displayed with an E value of 0.0) which both originated from tissues of neuroendocrine lung carcinoids (Cancer Genome Anatomy Project [20]). In comparison, searches with the sequences of H1.0 and H1.2 showed no clustering of EST entries in any library (data not shown). As a consequence of this result we decided to investigate the occurrence of H1x in NETs from the lung and the gastrointestinal tract by immunohistochemistry. Sections of poorly differentiated neuroendocrine carcinomas of lung and small intestine as well as of well-differentiated neuroendocrine carcinoma of pancreas were used (Table 1). Staining of these sections with affinity-purified H1x-antibody resulted in an intensive and uniform immunoreaction of the tumour cells (Figure 1). This result demonstrates that H1x is indeed highly expressed in NETs.

### Quantitative analysis

Since the immunohistochemical results showed a high expression of H1x in neuroendocrine tumour cells we next performed a quantitative analysis of the H1x expression on mRNA and on protein level in NET tissues compared with that in the corresponding normal tissue. Since several reports described a change of the expression level of the H1 replacement subtype H1.0 during tumourigenesis, the relative expression levels of this subtype was also determined.

**Figure 2:**
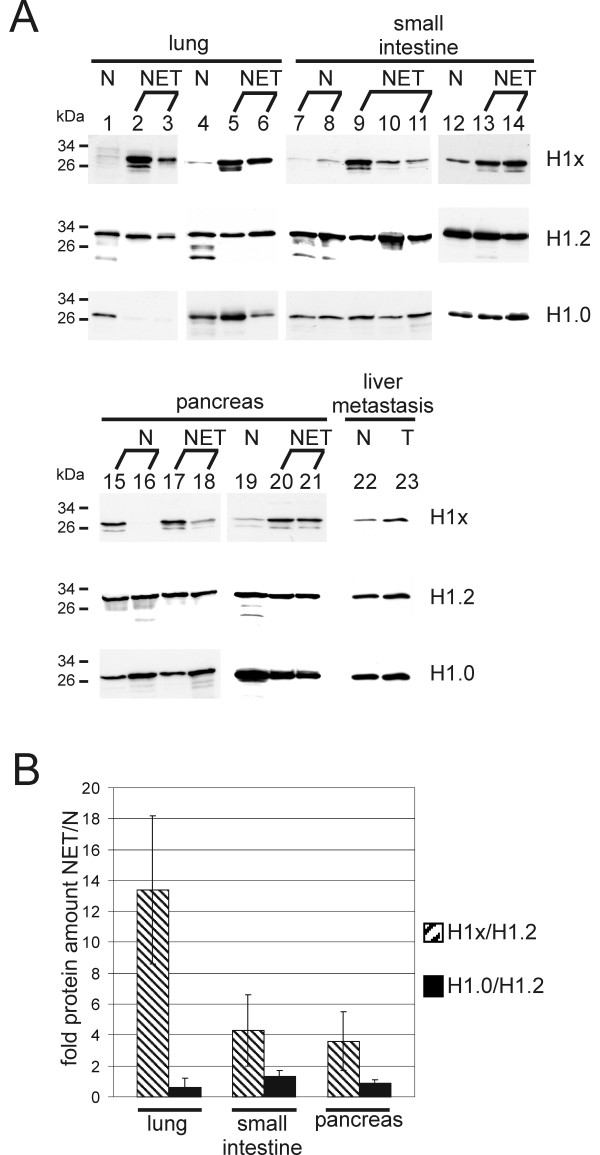
**(A) Relative protein amounts of the H1 histone subtypes H1x, H1.2 and H1.0 in non-neoplastic and neuroendocrine tumour tissues**. Determination of relative protein levels of histone H1x, main type subtype H1.2 and replacement histone H1.0 was done by Western blot analysis. Lane 1, normal tissue of lung (Table 1, sample no. 3), lane 2, neuroendocrine carcinoid of lung (no. 2), lane 3, poorly differentiated neuroendocrine small cell carcinoma of lung (no. 4), lane 4, normal tissue of lung (no. 5), lane 5, neuroendocrine carcinoid of lung (no. 6), lane 6, poorly differentiated small cell carcinoma of lung (no. 7), and lanes 7 and 8, normal tissue of small intestine (no. 8 and 9), lane 9, well-differentiated neuroendocrine carcinoma of small intestine (no. 10), lane 10, poorly differentiated neuroendocrine carcinoma (no. 11), and lane 11 well-differentiated neuroendocrine carcinoma of small intestine (no. 12), lane 12, normal tissue of small intestine (no. 8), lanes 13 and 14, neuroendocrine carcinoid of small intestine (no. 13 and 14), lane 15 and 16, normal tissue of pancreas (no. 15 and 16), lane 17, pancreatic neuroendocrine tumour of poor differentiation (no. 18) and lane 18, of good differentiation (no. 19), lane 19, normal tissue of pancreas (no. 16), lanes 20 and 21, well-differentiated carcinoma of pancreas (no. 17 and 20), lane 22, normal tissue of liver and lane 23, liver metastasis of well-differentiated carcinoma of pancreas (no. 21 and 22). (B) Densitometric quantitation of the immunoblots shown in (A). Ratios of H1x and H1.0 to H1.2 were calculated and standard deviations are indicated.

The determination of the relative protein amounts of H1x and H1.0 was done by immunoblotting. Histones were extracted by acid extraction from snap-frozen tumour material of neuroendocrine tumours and of the corresponding normal tissue. About equal amounts of H1 histones were loaded, as monitored by detection of the subtype H1.2 which is considered as one of the basal H1 subtypes detected in all cell types investigated so far [21,22]. In tissues from lung and small intestine a strikingly higher protein level of H1x was observed in the tumour tissues compared to the non-neoplastic tissues (Figures 2A/B, lung, small intestine). Regarding the pancreas, one of the non-neoplastic tissues showed a relatively high protein level of H1x whereas in the second sample from normal tissue the amount of H1x was very low (Figure 2A, lanes 15 and 16). Even in a liver metastasis of a pancreatic well-differentiated carcinoma, the level of H1x was higher than in non-neoplastic liver tissue (Figure 2A, lanes 22 and 23). In conclusion the H1x level was higher in the NET tissues than in the normal tissues (Figure 2B). In contrast, no increase in H1.0 protein level in NETs compared to non-neoplastic tissues was observed (Figure 2A and 2B, H1.0). The amount of H1.0 in the NET tissues was partially lower (e.g. Figure 2A, lanes 2 and 3) and partially higher (e.g. Figure 2A, lanes 5 and 14) than in the corresponding normal tissues. This observation fits the result of earlier studies which either found an increase or a decrease of H1.0 in tumour tissues in comparison to the paired normal tissues (reviewed in [14]).

**Figure 3:**
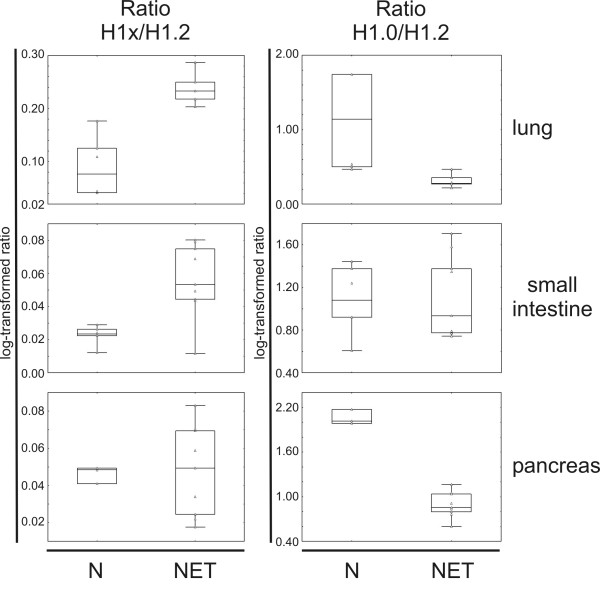
**Box plot presentation of the relative RNA levels of H1x and H1.0 determined by quantitative real-time RT-PCR**. Total RNA was extracted from normal lung tissues (Table 1, samples no. 1 and 3), NETs from lung (no. 2 and 4), normal tissues from small intestine (no. 8 and 9), NETs from small intestine (no. 10, 11 and 12), normal tissue from pancreas (no. 15) and pancreatic NETs (no. 17, 18 and 19) and used for reverse transcription. The generated cDNA from each patient was used for triplicate quantitative real-time PCR. Gene expression levels of *H1x *and *H1.0 *were determined by relative quantification using the expression of histone *H1.2 *for normalization. Wilcoxon test was used for statistical analysis. The mRNA level of H1x was significantly increased in NET tissue of lung (*p *= 0.004) and small intestine (*p *= 0.01). Horizontal line, median; inner box, 25 and 75% interval; outer spread, minimum and maximum.

The mRNA level of H1x, H1.2 and H1.0 in NET tissues and non-neoplastic tissues was analysed by quantitative real-time RT-PCR. The ratios of H1x and H1.0 to the main-class subtype H1.2 were calculated (Figure 3). A significantly higher expression of H1x in the NETs of lung and small intestine compared to the non-neoplastic tissues was found. However, in the tissue samples of pancreas no difference in the H1x expression but a decrease of the H1.0 expression was observed. Since high protein amounts of H1x have been determined in the NETs of pancreas by immunohistochemistry and Western blot analysis, this result may be explained by a differential protein turnover or varied kinetics of transcription and translation like is has been described for the subtype H1.0 [23].

In summary, although the sample size of the present study was relatively low, these results clearly show an increased expression of the H1 histone subtype H1x in the analysed group of NETs compared to the corresponding normal tissues.

### Detection of H1x in neuroendocrine cells

**Figure 4:**
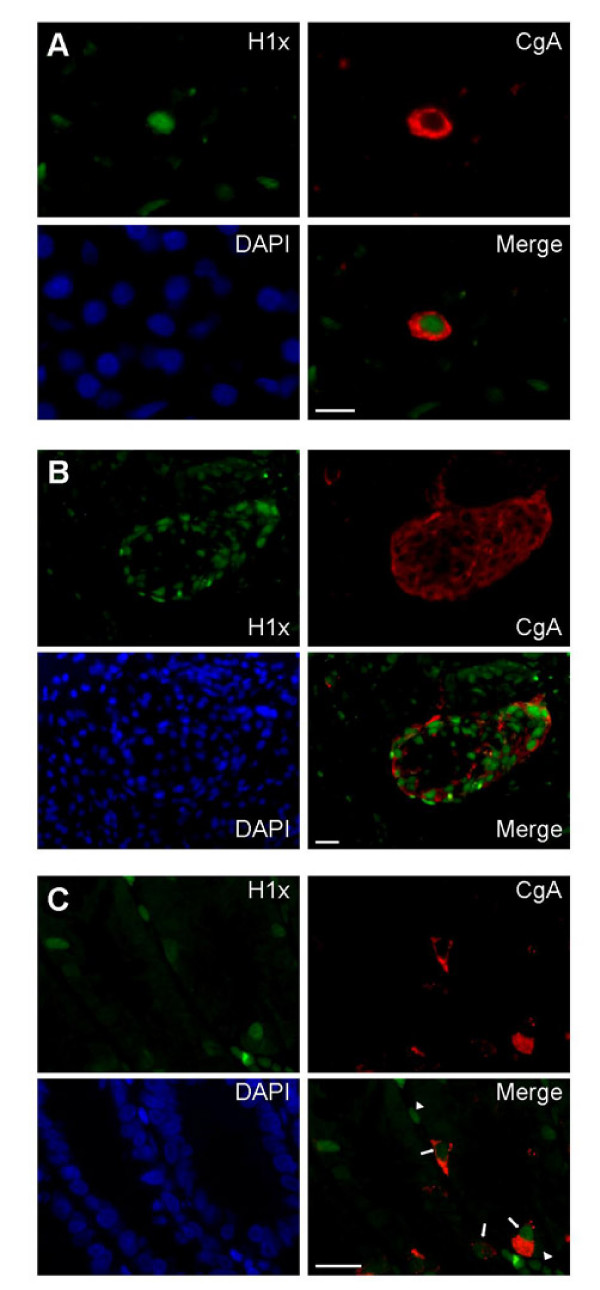
**Indirect immunofluorescence detection of H1x and chromogranin A in sections of paraffin wax-embedded samples from pancreas and small intestine**. Chromogranin A (CgA) was used as a marker protein for neuroendocrine cells which show scattered location in these tissues. (A) Chromogranin A-positive cells in pancreatic tissue exhibit a relatively high expression of H1x. Scale bar = 10 μm. (B) Cells of a islet of Langerhans show a strong immunoreaction with the anti-H1x antibody. Scale bar = 20 μm. (C) H1x is abundant in chromogranin A-positive cells of small intestine (arrows) compared to the surrounding cells of the glandulae intestinales and also in some cells of the stroma of the villi (arrowheads). Scale bar = 20 μm.

The finding that H1x is highly expressed in neuroendocrine tumour cells raised the question whether H1x is generally abundant in neuroendocrine cells. To answer this question, indirect immunofluorescence double-labelling of H1x and chromogranin A was performed. Chromogranin A, a marker protein for neuroendocrine cells, is a secretory granin protein that is co-stored and co-released with peptides and amines. It seems to be crucial for the formation of secretory granules and sequestration of hormones in neuroendocrine cells [24-26]. In pancreatic tissue, scattered chromogranin A-positive cells show a high protein amount of histone H1x compared to the surrounding chromogranin A-negative cells (Figure 4A). Interestingly, endocrine cells of islets of Langerhans also exhibit a relatively high expression of H1x (Figure 4B). Double-labelling of sections of small intestine revealed that the chromogranin A-positive neuroendocrine cells contain higher levels of H1x than the surrounding cells of the glandulae intestinales (Figure 4C). However, an abundance of H1x was also observed in some cells of the stroma of the villi. Unfortunalely, analysis of lung tissue with immunofluorescence labelling was not possible, since lung sections display a very strong autofluorescence which is mainly caused by elastin fibers [27,28].

## Conclusion

The results of this study clearly show that the H1 subtype H1x is considerably expressed in neuroendocrine cells of pancreas and small intestine, and also in endocrine cells of islets of Langerhans. Thus, the high expression of histone H1x in NETs is probably due to the high expression of this protein in the cells from which these tumours originated. Further research into molecular mechanisms by which H1x exerts its function is needed to find out whether this increased expression is linked to the endocrine function of these cells and/or whether this H1 subtype participates in the tumourigenesis of NETs.

## Competing interests

The authors declare that they have no competing interests.

## Authors' contributions

JW performed the immunohistochemistry, designed the primers, carried out the quantitative real-time RT-PCR, participated in the Western blot analysis and corrected the manuscript. FH participated in the primer design, the quantitative real-time PCR, performed the statistical analysis, helped with the immunohistochemistry and critical read the manuscript. OH and BCD provided clinical expertise, clinical samples and critical reading of the manuscript. LF performed the pathological evaluation, participated in the study design and corrected the draft. DD discussed the results, helped with the interpretation of the data and corrected the draft. NH conceived the study, carried out the Western blot analysis, performed the double-labelling experiments and drafted the manuscript. All authors read and approved the final manuscript.

## Pre-publication history

The pre-publication history for this paper can be accessed here:



## References

[B1] Luger K, Mader AW, Richmond RK, Sargent DF, Richmond TJ (1997). Crystal structure of the nucleosome core particle at 2.8 A resolution. Nature.

[B2] Kornberg RD, Lorch Y (1999). Twenty-five years of the nucleosome, fundamental particle of the eukaryote chromosome. Cell.

[B3] Ausio J (2006). Histone variants – the structure behind the function. Brief Funct Genomic Proteomic.

[B4] Happel N, Schulze E, Doenecke D (2005). Characterisation of human histone H1x. Biol Chem.

[B5] Stoldt S, Wenzel D, Schulze E, Doenecke D, Happel N (2007). G1 phase-dependent nucleolar accumulation of human histone H1x. Biol Cell.

[B6] Brown DT (2003). Histone H1 and the dynamic regulation of chromatin function. Biochem Cell Biol.

[B7] Alexandrow MG, Hamlin JL (2005). Chromatin decondensation in S-phase involves recruitment of Cdk2 by Cdc45 and histone H1 phosphorylation. J Cell Biol.

[B8] Downs JA, Kosmidou E, Morgan A, Jackson SP (2003). Suppression of homologous recombination by the Saccharomyces cerevisiae linker histone. Mol Cell.

[B9] Ducasse M, Brown MA (2006). Epigenetic aberrations and cancer. Mol Cancer.

[B10] Santos-Reboucas CB, Pimentel MM (2007). Implication of abnormal epigenetic patterns for human diseases. Eur J Hum Genet.

[B11] Tan KB, Borun TW, Charpentier R, Cristofalo VJ, Croce CM (1982). Normal and neoplastic human cells have different histone H1 compositions. J Biol Chem.

[B12] Mannironi C, Rossi V, Biondi A, Ubezio P, Giudici G, Masera G, D'Incalci M (1988). Comparison of histone variant synthesis in human lymphocytic leukemia cells and in normal lymphocytes. Cancer Res.

[B13] Goodlad GA, Clark CM (1995). H1 histone subtype distribution and DNA topoisomerase activity in skeletal muscle of tumour-bearing rats. Cancer Lett.

[B14] Zlatanova J, Doenecke D (1994). Histone H1 zero: a major player in cell differentiation?. Faseb J.

[B15] Kostova NN, Srebreva LN, Milev AD, Bogdanova OG, Rundquist I, Lindner HH, Markov DV (2005). Immunohistochemical demonstration of histone H1(0) in human breast carcinoma. Histochem Cell Biol.

[B16] Klöppel G, Perren A, Heitz PU (2004). The gastroenteropancreatic neuroendocrine cell system and its tumors: the WHO classification. Ann N Y Acad Sci.

[B17] Grötzinger C (2004). Tumour biology of gastroenteropancreatic neuroendocrine tumours. Neuroendocrinology.

[B18] Ferolla P, Faggiano A, Avenia N, Milone F, Masone S, Giampaglia F, Puma F, Daddi G, Angeletti G, Lombardi G, Santeusanio F, Colao A (2007). Epidemiology of non-gastroenteropancreatic (neuro)endocrine tumours. Clin Endocrinol (Oxf).

[B19] Livak KJ, Schmittgen TD (2001). Analysis of relative gene expression data using real-time quantitative PCR and the 2(-Delta Delta C(T)) Method. Methods.

[B20] Krizman DB, Wagner L, Lash A, Strausberg RL, Emmert-Buck MR (1999). The Cancer Genome Anatomy Project: EST sequencing and the genetics of cancer progression. Neoplasia.

[B21] Meergans T, Albig W, Doenecke D (1997). Varied expression patterns of human H1 histone genes in different cell lines. DNA Cell Biol.

[B22] Parseghian MH, Hamkalo BA (2001). A compendium of the histone H1 family of somatic subtypes: an elusive cast of characters and their characteristics. Biochem Cell Biol.

[B23] Rousseau D (1996). Uncoordinated regulation of histone H1(0)synthesis and H1(0) mRNA level. Biochem Mol Biol Int.

[B24] Winkler H, Fischer-Colbrie R (1992). The chromogranins A and B: the first 25 years and future perspectives. Neuroscience.

[B25] Lamberts SW, Hofland LJ, Nobels FR (2001). Neuroendocrine tumor markers. Front Neuroendocrinol.

[B26] Taupenot L, Harper KL, O'Connor DT (2003). The chromogranin-secretogranin family. N Engl J Med.

[B27] Mauderly JL, Bice DE, Cheng YS, Gillett NA, Henderson RF, Pickrell JA, Wolff RK (1989). Influence of experimental pulmonary emphysema on the toxicological effects from inhaled nitrogen dioxide and diesel exhaust. Res Rep Health Eff Inst.

[B28] Deyl Z, Macek K, Adam M, Vancikova O (1980). Studies on the chemical nature of elastin fluorescence. Biochim Biophys Acta.

